# Pigeon Pea Husk for Removal of Emerging Contaminants Trimethoprim and Atenolol from Water

**DOI:** 10.3390/molecules26113158

**Published:** 2021-05-25

**Authors:** Severin Eder, Manuel Torko, Alessia Montalbetti, Paride Azzari, Laura Nyström

**Affiliations:** 1 Laboratory of Food Biochemistry, Institute of Food, Nutrition and Health, Department of Health Science and Technology, ETH Zurich, Schmelzbergstrasse 9, 8092 Zurich, Switzerland; severin.eder@hest.ethz.ch (S.E.); mtorko@student.ethz.ch (M.T.); malessia@student.ethz.ch (A.M.); 2 Laboratory of Food and Soft Materials, Institute of Food, Nutrition and Health, Department of Health Science and Technology, ETH Zurich, Schmelzbergstrasse 9, 8092 Zurich, Switzerland; paride.azzari@hest.ethz.ch

**Keywords:** low-cost adsorbent, side streams, pigeon pea, adsorption, emerging contaminants, trimethoprim, atenolol, adsorption kinetics, adsorption thermodynamics

## Abstract

The pace of industrialization and rapid population growth in countries such as India entail an increased input of industrial and sanitary organic micropollutants, the so-called emerging contaminants (EC), into the environment. The emission of EC, such as pharmaceuticals, reaching Indian water bodies causes a detrimental effect on aquatic life and ultimately on human health. However, the financial burden of expanding sophisticated water treatment capacities renders complementary, cost-efficient alternatives, such as adsorption, attractive. Here we show the merits of washed and milled pigeon pea husk (PPH) as low-cost adsorbent for the removal of the EC trimethoprim (TMP) and atenolol (ATN) that are among the most detected pharmaceuticals in Indian waters. We found a linear increase in adsorption capacity of PPH for TMP and ATN at concentrations ranging from 10 to 200 μg/L and from 50 to 400 μg/L, respectively, reflecting the concentrations occurring in Indian water bodies. Investigation of adsorption kinetics using the external mass transfer model (EMTM) revealed that film diffusion resistance governed the adsorption process of TMP or ATN onto PPH. Moreover, analysis of the adsorption performance of PPH across an extensive range of pH and temperature illustrated that the highest adsorption loadings achieved concurred with actual conditions of Indian waters. We anticipate our work as starting point towards the development of a feasible adsorbent system aiming at low-cost water treatment.

## 1. Introduction

Worldwide access to clean water poses one of the greatest global challenges amidst a rapidly growing world population [1]. This endeavor becomes additionally complex due to the continuous deterioration of water quality caused by intensified anthropogenic activities in light of an increasing population [2]. Rapid industrialization, agricultural growth, and unstructured urbanization cause the entry of untreated sanitary waste, industrial effluents, and runoff from agricultural lands into water bodies and thus their contamination [1,3]. In this context, a broad group of organic micropollutants, the so-called emerging contaminants (EC), which were historically considered to be of minor concern given their distribution and concentration, have recently received particular attention. EC encompass substances belonging to the groups of pesticides, pharmaceuticals, personal care products, surfactants, and phthalates [4,5]. With the intensification of human activities and advances in analytical techniques, there is increasing awareness of EC and their adverse effects on the environment and human health.

India, as the fifth largest producer of pharmaceuticals and being among the largest consumer markets of drugs, faces significant issues with environmental inputs of pharmaceuticals, a major subgroup of EC [5,6]. Atenolol (ATN) and trimethoprim (TMP) represent one of the most frequently prescribed antihypertensives and antibiotics, respectively, and are among the most detected medical compounds in Indian water treatment plants. Concentrations in the range from 0.2 to 41.4 μg/L for ATN and from 0.003 to 4 μg/L for TMP have been reported in Indian water bodies [5,6]. Thus, the environmentally relevant concentration of both substances (>1 μg/L) is exceeded by multiple times in certain areas [7,8]. Their depositions into water reserves cause ecotoxicological effects on aquatic life and trigger antibiotic resistance in pathogens, which eventually presents a serious threat to human health [6,9].

However, the capacity of water treatment in India corresponds only to one third of the volumes of wastewater generated from its population. Moreover, the existing technology and infrastructure for water purification demonstrates rather limited effectiveness for the removal of pharmaceutical residues [4,5,6]. Yet, the nationwide extension with high-technology systems is constrained due to immense investment expenses [1]. In this regard, adsorption presents an accessible, cost-efficient addition or alternative owing to the ease of operation and simplicity of design [2,10].

Recently, the replacement of activated carbon, the industrial standard, with cheaper alternatives based on agro-industrial side streams, the so-called low-cost adsorbents, has been pursued [2]. These waste materials possess a variety of functional groups and contribute to a reduction in unutilized plant by-products, thereby rendering their use attractive from several perspectives [11]. This vision has already been realized with the launch of the first household product to facilitate access to clean water in the Indian market. The Tata Swach system is an example of a successful application of low-cost adsorbents in a water purification device. The full-fledged, portable device, relies on rice husk ash as the adsorbent, and enables safe drinking water, in India, for low income households at an affordable price [12]. The establishment of a sustained, viable low-cost concept requires that a side stream material is readily available locally and in sufficient quantity. Pigeon Pea (*Cajanus cajan*) is the second most cultivated pulse in India and is mainly consumed as “Dal” [13,14]. For this, pigeon peas are dehulled and the cotyledon is split by an industrial process [11]. The production volume, in 2016, accounted for 2.8 Mt in India, of which around 8%, or approximately 230,000 t, account for husk material that end as side stream [11,14]. Consequently, pigeon pea husk (PPH) has potential to be utilized as a low-cost adsorbent; therefore, enhancing its exploitation. To date, adsorption studies with PPH have been limited to successful application for adsorption of various metals [11,15,16,17]. In contrast, the utilization of PPH for EC, such as pharmaceuticals, is still untapped.

Furthermore, many previous works included inconsistencies in their methodological approach and have used invalid assumptions for the process considered [18]. Numerous existing studies have based their investigations of adsorption thermodynamics on incorrect computations. An inappropriate derivation of the thermodynamic equilibrium constant *K_eq_* oftentimes results in unreasonable thermodynamic parameters [19]. Furthermore, the selection of isotherm models to describe the experimental isotherm data frequently relies on statistically incorrect criteria [20]. However, a valid modeling of the equilibrium data is essential for a meaningful evaluation of the adsorption process and the proper deduction of the adsorption isotherm parameters [10]. Moreover, the elucidation of adsorption kinetics is commonly performed by models that originate from the description of chemical reaction kinetics [21]. Therefore, these models fail to facilitate conclusive insight into the actual driving mechanisms of the mass transfer observed. The selection of adequate kinetic models with consideration of the characteristics of the adsorbent and the adsorption process is indispensable for a comprehensive assessment of the adsorption kinetics.

In this study, we evaluated the suitability of PPH as a low-cost adsorbent for the removal of the pharmaceutical substances, TMP and ATN, both present at elevated concentrations in Indian water bodies. For this purpose, we conducted a comprehensive analysis of the adsorption processes in a batch adsorption design which encompassed: (i) interpretation of the adsorption equilibrium, (ii) a detailed description of the adsorption kinetics, (iii) investigations of the thermodynamic parameters, and (iv) elucidation of the effects of temperature and pH on the adsorption performance. Particular attention was paid to the accurate derivation of the dimensionless thermodynamic equilibrium constants in order to obtain reasonable inferences about the nature of the adsorption process. In addition, we focused on numerical simulations of the adsorption kinetics with the external mass transfer model (EMTM) to validate the governing mechanism of the mass transfer. Furthermore, the selection of the appropriate isotherm model to predict the equilibrium data was based on the Akaike information criterion (AIC) methodology that provides a statistical criterion for the adequacy of considered candidate models. This study demonstrates that PPH poses a promising asset as a low-cost adsorbent for the removal of TMP and ATN and provides the scientific framework for the development of adsorption systems intended for EC with similar physiochemical properties.

## 2. Results and Discussions

### 2.1. Surface Morphology and Physical Characteristics of Pigeon Pea Husk (PPH)

**Figure 1:**
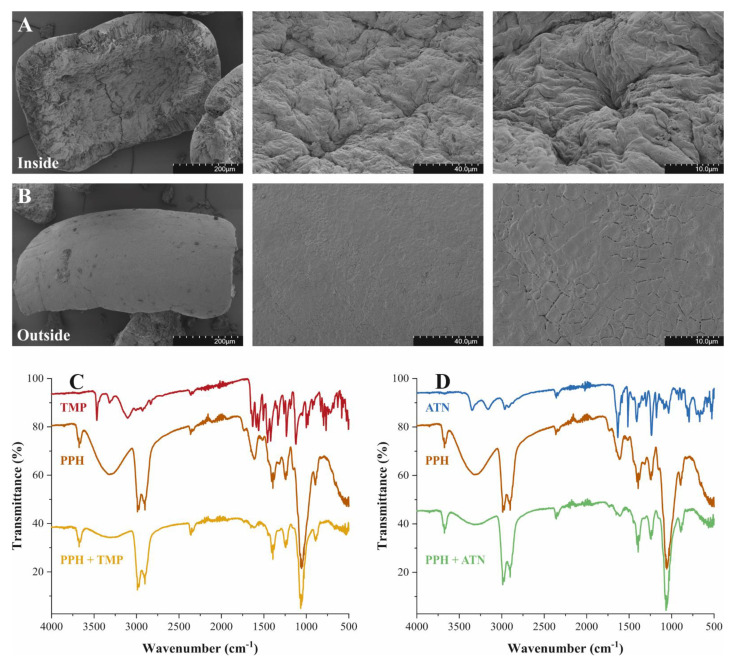
Representative SEM micrographs. (**A**) inside of PPH; (**B**) outside of PPH, at a series of magnifications. (**C**,**D**) FTIR spectra of TMP (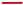), ATN (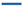), PPH (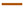) and after adsorption of TMP (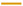) or ATN (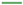) on PPH.

The micrographs acquired displayed a distinct difference in the surface morphology of the inner and outer surfaces of the pigeon pea husk (PPH) particles (Figure 1A,B); however, both sides were characterized by the regularity of their respective patterns. The SEM investigations of the inner surface demonstrated a nonporous, wrinkled surface (Figure 1A). The undulating morphology was devoid of sharp edges and enlarged the external surface area. In contrast, the outer surface exhibited a homogeneous, smooth surface (Figure 1B). As on the inner surface, pronounced cavities were absent on the outer surface except for smaller cracks distributed across the surface in a somewhat ordered geometry. Generally, the micrographs recorded provided no indication of the presence of a considerable internal surface within PPH particles. Nitrogen sorption and BET analysis confirmed the negligible internal surface area and suggested that adsorption occurs at the external surface (Table 1). Furthermore, the physical properties of PPH are summarized in Table 1. For more information on the particle size distribution of the PPH material, see Supplementary Materials (Figure S1).

### 2.2. Characterization of Surface Functionalities and Adsorption Process

Fourier-transform infrared (FTIR) spectroscopy was used to characterize the structural functionalities of PPH and to confirm the adsorption of trimethoprim (TMP) or atenolol (ATN) onto PPH. The evaluation focused on the functional group region of the spectra before and after TMP or ATN adsorption on PPH to identify the presence of characteristic functionalities (Figure 1C,D). The IR spectra of PPH (Figure 1C,D) exhibited bands typical for plant fiber material, such as the broad peak centered at 3320 cm^−1^, attributed to intermolecular H-bonded O–H stretching vibrations of alcoholic groups in polysaccharides [22,23]. The medium, sharper band detected at 3675 cm^−1^ can be attributed to O–H stretching vibrations of free alcohol groups. Furthermore, the signals from 2990 to 2880 cm^−1^ originated from C–H stretch vibrations. The peak detected at 1740 cm^−1^ can be assigned to C=O stretching vibrations of carboxyl group functionalities. Moreover, the bands at 1620 and 1400 cm^−1^ are typically ascribed to the stretching of carboxyl groups (O=C–O) [23,24]. The peak, occurring at 2360 cm^−1^, originated from the presence of trace amounts of atmospheric CO_2_ during the measurement.

The spectra acquired for TMP (Figure 1C) exhibited bands characteristics for its structural features [25,26]. The sharp bands at 3470 and 3320 cm^−1^ were assigned to N–H stretching vibration of primary aromatic amine groups. Furthermore, the shoulder peak centered at 3110 cm^−1^ corresponded to the N–H bending vibration [22,25]. The bands in the region 3030–2810 cm^−1^ were attributed to C–H stretch vibrations of the pyrimidine, benzyl, and methyl groups [26]. Moreover, signals ranging from 1670 to 1540 cm^−1^ and at 1510 cm^−1^ correlated to the aromatic ring stretch of C=C vibrations. Bands from 1360 to 1210 cm^−1^ and the broad peak at 1130 cm^−1^ were ascribed to C–N stretching vibrations of the aromatic amine group and the C–O stretching vibrations of ester moieties, respectively [22,26].

Similarly, ATN showed characteristic bands corresponding to its structural features (Figure 1D). The two peaks at 3350 and 3160 cm^−1^ corresponded to the N–H stretching vibration of N–H_2_ groups. It may be assumed that an expected band in the region from 3360 to 3310 cm^−1^, related to a secondary amine functionality, probably overlaps with the strong N–H stretching signal [22]. Bands in the region 2990–2820 cm^−1^ originated from alkyl C–H stretch vibrations and the strong peak at 1635 cm^−1^ was attributed to H-bonded C=O stretching vibration of a primary amid group. Additionally, bands from 1620 to 1580 cm^−1^ and the sharp signal at 1515 cm^−1^ were characteristic for the aromatic ring stretch of C=C vibrations. Bands at 1415 and 1240 cm^−1^ were ascribed to the O–H bending of the alcohol and C–O stretching vibrations of the phenolic ester group, respectively [22].

A comparison before and after adsorption of TMP or ATN on PPH revealed an alteration or introduction of characteristic signals in the PPH spectra (Figure 1C,D). For instance, the broad signal of O–H stretching vibrations at 3320 cm^−1^, noticeable in the PPH spectra, extenuated remarkably after adsorption of TMP. This attenuation might indicate the involvement of O–H moieties, present on the PPH, and aromatic amine groups, as part of the TMP structure, in the adsorption process. Additionally, the peak at 1450 cm^−1^, initially slightly pronounced in the PPH spectra, presented a characteristic signal after the adsorption experiment. Moreover, the bands ranging from 1690 to 1570 cm^−1^ significantly flattened in the spectra of PPH with adsorbed TMP as compared with the PPH spectra. Furthermore, the “fingerprint region” <1000 cm^−1^ exhibited considerable variations, illustrating the presence of newly introduced functionalities by TMP on PPH (Figure 1C). The signal intensities at higher wave numbers decreased within the PPH spectra after ATN adsorption, as observed for TMP, suggesting that the corresponding functionalities participated in the adsorption (Figure 1D). A similar observation was made for the signals present in the PPH spectra ranging from 1700 to 1570 cm^−1^, indicating the presence of ATN on PPH after adsorption. The differentiation of the “fingerprint region” <1000 cm^−1^ within the PPH spectra after the adsorption experiment corroborated the adsorption of ATN. The FTIR investigations of PPH before and after exposure to TMP or ATN revealed the accumulation of the respective substance on PPH and verified a successful adsorption process.

### 2.3. Adsorption Isotherm and Saturation Limit

**Figure 2:**
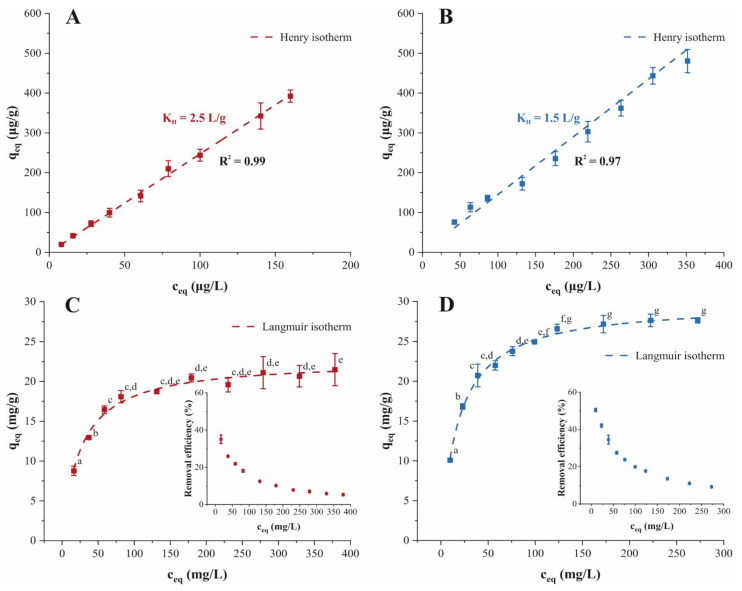
Experimental data (■) and Henry isotherm model (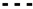) of the adsorption isotherms for (**A**) TMP (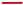) and (**B**) ATN adsorption (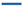) on PPH in the ppb concentration range. Experimental data (■) and Langmuir isotherm model (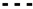) of the adsorption isotherms for (**C**) TMP (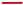) and (**D**) ATN adsorption (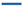) on PPH in the ppm concentration range. Different letters denote significant differences between isotherm points (*p* < 0.05, *n* = 3).

The adsorption equilibrium isotherms represented the adsorbed amount of TMP or ATN on PPH relative to the concentration of TMP or ATN in solution at equilibrium (Figure 2). Equilibrium experiments on the adsorption of TMP or ATN were conducted once in the ppb and once in the ppm concentration range. The adsorption isotherms acquired in the lower ppb range revealed insights into the adsorption process at TMP and ATN concentrations occurring in Indian water bodies (Figure 2A,B). Both adsorption systems were characterized by a linear relationship between PPH loading and TMP or ATN concentration. The Henry isotherm, suited for linear isotherms and commonly found at low concentrations, fitted the experimental data well (R^2^ > 0.97) (Figure 2A,B). The Henry constant, *K_H_*, reflects the proportionality between the adsorbent loading and the concentration of TMP or ATN in solution. For both systems, the resulting *K_H_* values were greater than 1 L/g, illustrating a steeper increase in adsorption uptake with respect to a change in concentration.

The adsorption isotherm at a higher ppm concentration range enabled the determination of the maximum adsorption capacity of TMP and ATN on PPH (Figure 2C,D). The shape of the equilibrium isotherm reflects the affinity between adsorbate and adsorbent and facilitates insight into a possible adsorption mechanism associated with the interaction. According to the classification for liquid-solid adsorption systems, both adsorption isotherms observed can be identified as L curves [27]. The concave curves follow the assumption that a higher concentration of TMP or ATN results in higher adsorption capacity until the number of available adsorption sites become scarce. The increasing competition between TMP or ATN molecules for a vacant site results in the progressive saturation of PPH. Moreover, the L curve isotherm suggests that the interactions between TMP or ATN and PPH, respectively, are driven by relatively weak forces.

We applied the Langmuir, Freundlich, Sips, Tóth, and Redlich-Peterson isotherm models to the equilibrium data of TMP and ATN adsorption to gain detailed insight into the nature of the adsorption process. According to the Akaike information criterion (AIC) methodology [28], we obtained a statistical comparison to determine the best model for the approximation of the experimental data. The isotherm model resulting in the highest *w_i_* value and lowest Δ*_i_* value was deemed to be the best fit. The Langmuir isotherm presented the best approximation for TMP and ATN adsorption onto PPH, respectively (Table 2). Considering the AIC_c_ values and statistical parameters derived thereof among models studied, the prediction of the experimental data for TMP adsorption ranked as follows: Langmuir > Tóth > Sips > Redlich-Peterson > Freundlich. In the case of ATN adsorption on PPH, the isotherm models ranked as follows: Langmuir > Tóth/Sips > Redlich-Peterson > Freundlich. Generally, isotherm models incorporating the saturation effect at high adsorbate concentration provided better fits than models approaching the Freundlich model. The Δ*_i_* facilitates a relative strength of evidence comparison among candidate models and can be assessed following some simple rules, i.e., values of Δ*_i_* ≤ 2 indicate considerable evidence for the model, models having 4 ≤ Δ*_i_* ≤ 7 indicate substantially less support, and models with Δ*_i_* > 10 have basically no evidence relative to the best model [28]. According to these guidelines, the Freundlich model possessed no evidence as a plausible model fit for either the adsorption of TMP (Δ*_i_* = 26) or ATN (Δ*_i_* = 56) onto PPH (Table 2). However, there was considerable strength of evidence for the Redlich-Peterson (Δ*_i_* = 2.5), Sips (Δ*_i_* = 1.8), and Tóth (Δ*_i_* = 1.7) isotherm models for TMP adsorption on PPH. Likewise, the three-parameter isotherms, Redlich-Peterson (Δ*_i_* = 2.5), Sips (Δ*_i_* = 2.4), and Tóth (Δ*_i_* = 2.4) showed substantial support as adequate predictions of the empirical data for ATN adsorption. The Akaike weights, *w_i_*, provide a measure of the “weight of evidence”, interpretable as probabilities, that a given model is indeed the best for the data obtained. The Langmuir model presented *w_i_* values of 0.43 and 0.57 for TMP or ATN adsorption on PPH, expressible as 43% and 57% confidence in being the best approximation, respectively (Table 2). Owing to the high *w_i_* values, any possible model selection uncertainty could be eliminated. On the basis of Δ*_i_*, the evidence ratio (*ER*) can be derived as an additional measure to reflect the relative likelihood between models. For the adsorption of TMP on PPH, the *ER* obtained suggested that the Langmuir model was over 46 × 10^3^ times more likely than the Freundlich model, two times more likely than the Sips and Tóth model, and three times more likely than the Redlich-Peterson model (Table 2). Accordingly, it was observed that the best-fitting Langmuir model was 1.2 × 10^12^ times more likely than the Freundlich model, and three times more likely than the Sips, Tóth, and Redlich-Peterson models in the case of ATN adsorption on PPH (Table 2). Overall, the Freundlich model yielded poor model fits (*ER* > 150) for the adsorption processes considered [28].

The Langmuir isotherm assumes the gradual filling of all possible adsorption sites without intermolecular interaction between adjacent adsorbate molecules, resulting in the formation of a monolayer on the solid surface [29,30]. The complete monolayer adsorption capacity, *q_mL_*, reflects the saturation plateau. The binding constant, *K_L_*, relates to the affinity of the adsorbate towards the adsorbent with increment of adsorbate. Hence, higher *K_L_* corresponds to a higher increase in adsorption capacity as *K_L_* reflects the initial slope of the equilibrium curve. The low *K_L_* values observed describe the smooth initial slope of the adsorption isotherm and reflect the rapid attainment of PPH saturation with increasing initial concentration of TMP and ATN (Table 2). Furthermore, the separation factor, *R_L_*, derived from the isotherm data, permits inferences whether the considered adsorption process is favorable (0 < *R_L_* < 1), unfavorable (*R_L_* > 1), or irreversible (*R_L_* = 0) [30]. The *R_L_* values obtained for TMP and ATN adsorption on PPH ranged between 0 and 1, confirming the favorable nature of the adsorption processes studied (Table 2). The maximum achievable adsorption loading obtained for TMP and ATN onto PPH were 22.7 mg/g and 29.8 mg/g, respectively (Table 2).

The comparison of the *q_mL_* values observed with adsorption isotherms of TMP or ATN approximated with the Langmuir isotherm model facilitated a meaningful interpretation of the experimental values. The adsorption capacity of PPH for TMP and ATN was higher than for other low-cost adsorbents and even outperformed granular activated carbon (Table 3). As anticipated, higher achievable adsorption capacities were reported for biomass-derived activated carbonaceous adsorbents or specific clay minerals intended for the removal of TMP and ATN (Table 3). The economic benefits and minimal resource demand of PPH outweighs the inferior adsorption capacity and highlights the excellent performance of PPH as a sustainable low-cost adsorbent for TMP and ATN. Moreover, PPH removed over 35% of TMP and 50% of ATN at the lowest initial concentrations (Figure 2C,D). At the highest initial concentration of 400 ppm TMP and 300 ppm ATN, PPH still exhibited removal efficiencies close to 10 and over 5%, respectively.

### 2.4. Adsorption Kinetics

The elucidation of the underlying mechanisms that determine the rate of adsorption are pivotal to the understanding of the mass transfer under investigation. The external mass transfer model (EMTM) used in this work assumed that film diffusion was the only rate-limiting mechanism for adsorption and omitted constraints due to intraparticle resistances [21,37]. The external mass transfer through the boundary layer is reflected by the external mass transfer coefficient or film diffusion coefficient *k_F_*. Optimal *k_F_* values were obtained by matching the numerical solution of the EMTM to the experimental decay curves.

**Figure 3:**
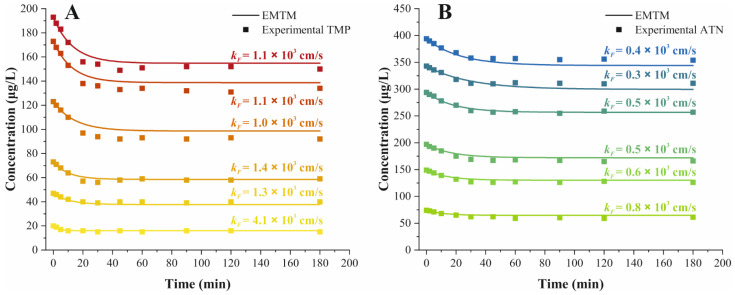
Experimental data (■) and prediction of (**A**) TMP (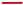) and (**B**) ATN (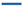) decay curves with the EMTM during adsorption on PPH at six initial concentrations, respectively.

The EMTM approximation of the experimental kinetic curves for six initial concentrations for TMP and ATN, ranging from 20 to 200 μg/L and from 75 to 400 μg/L, respectively, incorporated the optimal value of *k_F_* for each data set (Figure 3). The state of equilibrium was attained after 45 min for TMP adsorption and reached after 60 min for ATN adsorption on PPH (Figure 3). The EMTM predicted the kinetic data of TMP and ATN consistently, substantiating the governance of the adsorption rate only by film diffusion. Furthermore, N_2_ sorption experiments and BET analysis demonstrated negligible internal surface and corroborated the adequacy of the EMTM for the adsorption processes studied (Table 1).

The film diffusion coefficients, *k_F_*, observed for TMP and ATN, ranged from 4.1 to 1 × 10^−3^ cm/s and from 0.8 to 0.3 × 10^−3^ cm/s, respectively (Figure 3). Given the linear relationship of the equilibrium isotherm, the *k_F_* values for TMP and ATN were expected to remain constant across all initial concentrations studied. The experimental data are in line with this assumption, apart from the lowest initial concentration for both adsorbate substances (Figure 3). The deviations observed might arise due to the larger influence of experimental inaccuracies at lower concentrations. Constant *k_F_* values upon increasing initial concentration conform to the Henry isotherm theory, supposing that active sites on the adsorbent are available abundantly in relation to the adsorbate at sufficiently low concentrations. Accordingly, the external mass transfer resistances remained unchanged with increasing initial concentrations, as reflected in the *k_F_* values, within the concentration ranges considered for TMP and ATN.

Overall, film diffusion as the controlling mechanism of the mass transfers observed, suggests the opportunity to influence the adsorption rate by modification of adsorption conditions [37]. For instance, an increase in stirrer velocity of the batch system diminishes the thickness of the boundary layer and eventually reduces the equilibrium time required. Therefore, the rate of film diffusion, reflected in increasing *k_F_* values, would increase under these conditions. Generally, a reduction in the adsorbent’s particle size enhances the total surface area available for adsorption and further contributes to a reduction of the film diffusion resistance [37]. However, the adsorption process studied was not impeded due to constraints of the availability of sufficient adsorption sites, as previously discussed. Thus, any alteration of the particle size of PPH would be expected to result in an insignificant impact on the process. Ultimately, each intended application necessitates a critical assessment of benefits or disadvantages associated with the adsorption conditions considered to achieve an optimal compromise.

### 2.5. Effect of Temperature and Adsorption Thermodynamics

**Figure 4:**
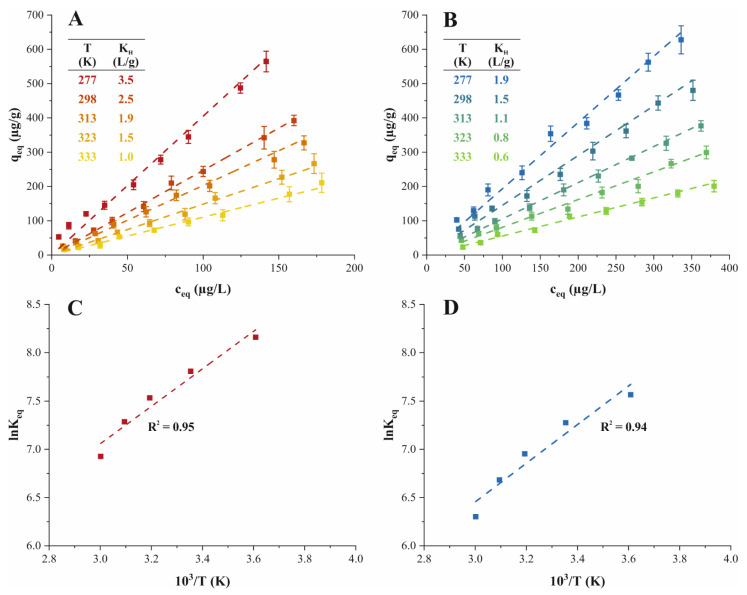
Experimental data (■) and Henry isotherm model (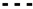) of the effect of temperature on the adsorption performance of (**A**) TMP (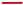) and (**B**) ATN (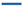) on PPH. Van’t Hoff plot and regression line (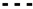) of (**C**) TMP (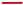) and (**D**) ATN (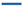) adsorption on PPH. Different letters denote significant differences between isotherm points (*p* < 0.05, *n* = 3).

Temperature substantially affects the molecular interactions during an adsorption process, and therefore impacts the resulting equilibrium state. The effect of temperature on TMP and ATN adsorption on PPH was monitored at five temperatures analogous to the adsorption isotherms in the ppb range, respectively (see Section 2.3). Both adsorption systems studied showed increasing adsorption capacities with decreasing temperature from 333 to 277 K, suggesting an exothermic adsorption process (Figure 4A,B). For instance, a temperature reduction from 333 to 277 K resulted in an increase in adsorption capacity from 211 ± 29 to 560 ± 40 μg/L for TMP, and from 201 ± 17 to 630 ± 50 μg/L for ATN adsorption on PPH at the highest initial concentration (Table S1). Overall, the adsorption capacity increased significantly from the highest to the lowest temperature for the respective initial concentrations of TMP or ATN considered (Figure 4A,B and Table S1). For a comprehensive overview with detailed information on significant differences between all temperature points for a respective initial concentration of TMP or ATN, see Supplementary Materials (Table S1).

The measurement of an adsorption isotherm at each temperature considered facilitated a meaningful derivation of the thermodynamic equilibrium constant *K_eq_* and a reliable elucidation of adsorption thermodynamics. The relevant thermodynamic parameters (Table 4) to assess the energetic conditions of the equilibrium states of the adsorption processes studied were derived from the Van’t Hoff plot (Figure 4C,D). The negative standard free Gibbs energy (Δ*G*°) values obtained indicated an exergonic process and showed the thermodynamically spontaneous nature of the adsorption (Table 4). Hence, the adsorption of TMP as well as ATN onto PPH presented a favorable process regardless of the solution temperature. Negative standard enthalpy (Δ*H*°) values evidenced the exothermic nature of both adsorption processes as anticipated based on the effect of temperature described above (Table 4). The Δ*H*° values observed for TMP and ATN adsorption suggested a physisorption controlled adsorption (Δ*H*° < 20 KJ) driven by Van der Waals forces as fundamental interaction force [29]. These findings are in line with the interpretation of the isotherm curve for the determination of the maximum adsorption capacity (see Section 2.3). Considering the standard entropy (Δ*S*°), positive values depicted the positive affinity of the adsorbents towards the adsorbate and the increase in disorder at the solid/liquid interface (Table 4). Furthermore, the dominant contribution of Δ*H*°, as compared with *T*Δ*S*°, to the negative Δ*G*° values observed, indicated that both TMP and ATN adsorption on PPH were enthalpy governed processes [29,38].

### 2.6. Effect of pH on Adsorption Performance

**Figure 5:**
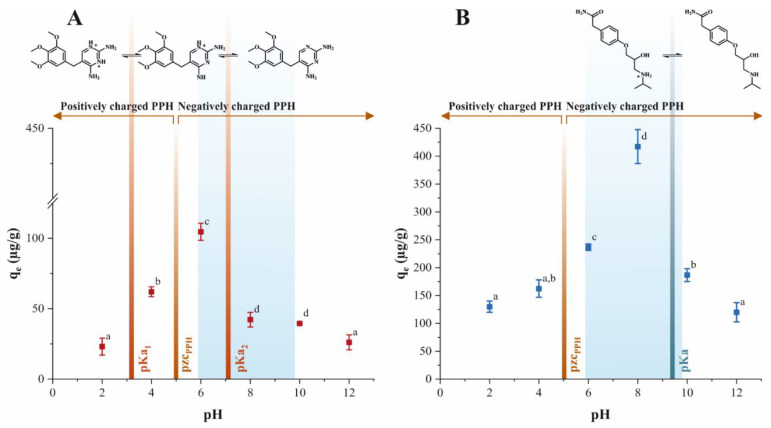
Effect of solution pH on the adsorption performance of (**A**) TMP (**■**) and (**B**) ATN (■) on PPH. The pH range of Indian water bodies (pH 5.9–9.8) is highlighted with the shadowed background. Different letters denote significant differences between experimental points (*p* < 0.05, *n* = 3).

The adsorption behavior and the resulting adsorption capacity depend decisively on the pH of the solution (Figure 5A,B). Investigations of the effect of pH of the aqueous solution were performed at RT with 50 μg/L initial TMP and 150 μg/L initial ATN concentrations, respectively. As expected, the data obtained presented a strong dependance of initial solution pH and the adsorption loading of TMP or ATN on PPH (Figure 5A,B). The highest adsorption performance for TMP was observed at pH 6 with 105 ± 7 μg/g (Figure 5A). In addition, PPH showed the maximum achievable loading for ATN at pH 8 yielding 420 ± 40 μg/g (Figure 5B). The adsorption capacities for both adsorbents decreased significantly at higher and lower pH conditions as compared with the respective point of maximum adsorption. In the case of TMP, the adsorption capacity achieved at pH 4 was significantly lower as compared with the maximum adsorption at pH 6, but nevertheless, significantly higher than in the remaining pH points (Figure 5A). Furthermore, no significant difference in adsorption capacity was observed between pH 8 and 10, whereas the adsorption loadings achieved at pH 2 and pH 12 were significantly lower than all other pH points studied for TMP. For adsorption of ATN on PPH, the data acquired at pH 6 showed significantly inferior adsorption capacity as compared with pH 8 but a significant increase towards the other pH points (Figure 5B). Moreover, the adsorption capacity of ATN at pH 10 deviated significantly from pH 2 and 12; however, no significant difference to pH 4 was detected. The adsorption capacity at pH 2, 4, and 12 did not vary significantly. The functionalities of adsorbent and adsorbate, reflected in the point of zero charge (pzc) and pK_a_ values, can explain the favorable or adverse interaction of adsorbent/adsorbate depending on the initial pH value (Figure 5A,B). TMP shows two pKa values (pK_a1_ = 3.2 and pK_a2_ = 7.1), corresponding to the pH values at which the deprotonation of the respective amino group occurs [39]. Hence, TMP is present in its TMP^2+^ state for pH < 3.2, in its TMP^+^ state for 3.2 < pH < 7.1, and as TMP for 7.1 < pH. Similarly, ATN occurs in is protonated state ATN^+^ below its pkA of 9.4 [40]. The pzc of PPH is defined by all functional groups on its surface and was determined to be at pH = 5 (Figure S2). At pH < pzc, the PPH carries a net positive charge and, at pH > pzc, the surface of PPH has a net negative charge.

The intersecting pH ranges where TMP or ATN exhibits a positive charge and PPH carries a negative surface charge corresponds to the pH points at which the highest adsorption charges were achieved, respectively. The effect of pH revealed the influence of PPH surface functionalities, which were elucidated within FTIR investigations, on the increase in attraction forces between adsorbate/adsorbent. Moreover, the pH values of the highest achievable adsorption loadings coincide with the pH range of Indian water bodies (pH 5.9–9.8) [41,42,43], substantiating PPH as an adequate low-cost adsorbent for the application studied (Figure 5A,B). Overall, the data acquired provides a strong starting point for broader applications of PPH as low-cost adsorbents targeted at the removal of pharmaceuticals or emerging contaminants with similar physicochemical properties within the pH range considered.

## 3. Materials and Methods

### 3.1. Chemicals

Trimethoprim (≥99%), atenolol (≥98%), formic acid (CH_2_O_2_, 98–100%), potassium bromide (KBr, >99%), and sodium hydroxide (NaOH, ≥98%) were obtained from Sigma-Aldrich (St. Louis, MO, USA). Hydrochloric acid (HCl, >37%) was purchased from VWR International (Radnor, PA, USA). Sodium chloride (NaCl, ≥99.5%) and acetonitrile (C_2_H_3_N, ≥99.9%) were purchased from Fisher Scientific (Waltham, MA, USA). All solutions were prepared with purified water using a Millipore MilliQ system (Billerica, MA, USA).

### 3.2. Preparation of Pigeon Pea Husk (PPH) Adsorbent Material

Pigeon pea husk (PPH) was provided by Bühler AG (Uzwil, Switzerland). The PPH was sieved through a stack of sieves, consisting of 4 and 2 mm mesh size, to remove unwanted material. Subsequently, the husk material was sieved with 1 mm mesh size to separate pigeon pea residues and smaller particles. Any remaining impurities were removed manually. Afterwards, the PPH was ground and sieved with a ZM200 ultra-centrifugal mill (Retsch GmbH, Haan, Germany) to obtain a particle size < 80 µm. The powdered PPH was washed to avoid leaching and discoloration of the solution during adsorption experiments. For this purpose, PPH was added to water in a 1:20 solid/solution ratio (*v*/*v*) and heated at 80 °C for 20 min under continuous agitation. After cooling, the suspension was centrifuged at 24 × 10^3^ g for 10 min in a Sorvall LYNX 4000 Superspeed Centrifuge (Thermo Fisher Scientific, Waltham, MA, USA). The colored supernatant was discarded, the PPH material was redissolved in water, and the procedure repeated five times in total. Eventually, the PPH was dried in a hot air oven at 60 °C overnight. PPH was stored in a desiccator under N_2_ atmosphere until further use.

### 3.3. Characterization of PPH and Functional Investigations

Micrographs of PPH were obtained using a SU5000 (Hitachi, Tokyo, Japan) high-resolution field emission scanning electron microscope (SEM). Second electron (SE) signal was acquired at 2.0 kV and a working distance of 5.5 mm. Samples were sputter coated with 4.5 nm platinum-palladium prior to the collection of micrographs. The particle size distribution of PPH powder was determined with a Partica LA-950 Laser Diffraction Particle Size Distribution Analyzer (Horiba, Kyoto, Japan). The solid density (*ρ_s_*) of the PPH was analyzed utilizing an AccuPyc 1330 He-Pycnometer (Micromeritics, Norcross, GA, USA). Hg-Porosimetry (Pascal 140/440 Hg-Porosimeter, Thermo Fisher Scientific, Waltham, MA, USA) was used to determine the apparent density (*ρ_p_*) of the PPH. The particle porosity (*ε_p_*) [44] of the PPH was calculated according to:

(1) $$\varepsilon_{p}=1-\frac{\rho_{p}}{\rho_{s}}.$$

Fourier-transform infrared (FTIR) spectroscopy analyses of PPH were conducted before and after adsorption experiments with TMP and ATN to infer about an effective adsorption. The FTIR spectra were recorded with a Jasco FT/IR-4100 spectrometer (JASCO Corporation, Tokyo, Japan) equipped with an Attenuated Total Reflectance (ATR) sampling accessory (ATR PRO ONE, JASCO Corporation, Tokyo, Japan) working with a monolithic diamond crystal. Spectra were recorded in the spectral range of 4500–500 cm^−1^ and processed using the Jasco Spectra Manager Version 2.05.02 (JASCO Corporation, Tokyo, Japan).

### 3.4. Batch Adsorption Design

The potential of PPH as an adsorbent for trimethoprim (TMP) or atenolol (ATN) was assessed with respect to the effect of initial adsorbent concentration, contact time, temperature, and pH. The batch adsorber design was chosen for the investigation of the adsorption systems, varying the parameter under investigation while all other parameters were kept constant. Equilibrium experiments were conducted at room temperature and neutral pH with an adsorbent dosage of 0.005 g, a solution volume of 0.05 L, and an initial concentration of TMP and ATN ranging from 10 to 200 μg/L and from 50 to 400 μg/L, respectively. The considered concentration ranges reflected a compromise between analytical viability and the simulation of residue concentrations occurring in Indian water bodies. The adsorption system was kept in contact for 180 min and constantly agitated at 300 rpm in a Unimax 1010 shaker (Heidolph, Schwabach, Germany). Adsorption kinetics were evaluated using six initial concentration for both adsorbates. The concentration decay over time was investigated with altered experimental conditions. PPH dose and solution volume were increased to 0.01 g and 0.1 L, respectively, to prevent distortion of the solid/liquid ratio due to aliquots retrieved at preset time points. The effect of temperature on the resulting adsorption capacities for TMP or ATN was analyzed over a temperature range from 4 to 60 °C. The effect of initial pH on the achievable adsorption loading was monitored over a pH range from 2 to 12. The pH of the solution was adjusted with HCl or NaOH at appropriate concentrations to minimalize volume distortion. Furthermore, equilibrium experiments were performed at higher concentration ranges of the adsorbates to determine the maximum achievable saturation limit of PPH for TMP or ATN. For this purpose, an adsorbent dose of 0.01 g, a solution volume of 0.01 L, and initial concentrations ranging from 25 to 400 mg/L for TMP and from 20 to 300 mg/L for ATN were selected.

After incubation, the mixtures were centrifuged for 15 min at 4000 rpm. Subsequently, the remaining TMP or ATN concentration in the liquid phase was determined using reversed-phase ultra-performance liquid chromatography with diode array detection (RP-UHPLC-DAD) (see Section 3.5). The adsorption uptake of TMP or ATN on PPH can be derived from the mass balance equation [30], which is given for any point of the adsorption process by:

(2) $$q_{t}=\frac{\left(c_{0}-c_{t}\right)V}{W},$$

where *c*_0_ and *c_t_* depict the initial adsorbate concentration in solution (μg/L or mg/L) and at any given time point (μg/L or mg/L), respectively; *q_t_* reflects the adsorbate concentration on PPH at given time point (μg/g or mg/g); *V* and *W* correspond to the volume of the solution (L) and the weight of PPH (g) used in the batch adsorption experiment, respectively.

### 3.5. Determination of Trimethoprim and Atenolol Concentration

The concentration of TMP and ATN in solution was analyzed by RP-UPLC-DAD. For the analysis of residual adsorbate concentration, an Agilent 1290 Infinity II System (Agilent Technologies, Santa Clara, CA, USA) equipped with an Acquity UPLC BEH C8 (2.1 × 100 mm, 1.7 μm) column operating at 30 °C was used. The method was adapted from the work by Krisko et al. [45], with slight modifications. The injection volume of the samples was 10 μL and a combination of the two mobile phases (A) water and (B) acetonitrile, containing 0.1% formic acid (*v*/*v*), was applied for elution at a flow rate of 0.4 mL/min. The resulting gradient program was 0–5 min, from 99.5% A and 0.5% B linear to 95% A and 5% B; 5–7 min, linear to 85% A and 15% B; 7–10 min, linear to 5% A and 95% B; 10–10.5 min, isocratic 5% A and 95% B; 10.5–11 min, linear to 95% A and 5% B; 11–12 min, linear to 99.5% A and 0.5% B. Detection of TMP and ATN was conducted at 272 nm. Mixtures of TMP and ATN at seven concentration levels of 0.01–0.25 mg/L and 0.05–0.4 mg/L, respectively, were used as external standards. For the determination of maximal adsorption loading, TMP and ATN at seven concentration levels of 0.25–20 mg/L and 0.5–50 mg/L, respectively, were used as external standards. Quantification was based on the external calibration method using the Chromeleon Chromatography Data System (CDS) Version 7 (Thermo Fisher Scientific, Waltham, MA, USA).

### 3.6. Adsorption Isotherm Models

The interpretation of the adsorption equilibrium presents an essential procedure in the investigation of a considered adsorption process. Linear equilibrium relationships were described using the Henry isotherm and fitted by linear regression to derive the isotherm parameter. The Henry isotherm is defined as:

(3) $$q_{eq}=K_{H}c_{eq},$$

where *q_eq_* and *c_eq_* are the equilibrium concentration in solution (μg/L) and on the adsorbent (μg/g), respectively. The equilibrium constant, known as the Henry constant K_H_ (L/g), equals the constant of proportionality, and reflects the relationship between liquid and solid phase concentration [29,30]. Equilibrium data, exhibiting a nonlinear course of the relationship, were analyzed using the Langmuir model, which is given by:

(4) $$q_{eq}=\frac{q_{mL}K_{L}c_{eq}}{1+K_{L}c_{eq}},$$

where *q_eq_* and *c_eq_* were defined earlier and *q_mL_* depicts the Langmuir constant related to the monolayer adsorption capacity (mg/g). The Langmuir constant *K_L_* reflects the affinity of binding sites and the free energy of sorption (L/mg) [29]. The nature of the Langmuir isotherm can be characterized by a dimensionless constant separation factor *R_L_*, which results from:

(5) $$R_{L}=\frac{1}{1+K_{L}c_{0}}$$

Specific values of *R_L_* can be attributed to the isotherm characteristics, being either favorable (0 < *R_L_* < 1), unfavorable (*R_L_* > 1), linear (*R_L_* = 1), or irreversible (*R_L_* = 0) [30]. Moreover, the Freundlich, Redlich Peterson, Sips, and Tóth isotherm models were applied to nonlinear adsorption isotherms. Here, the experimental data was fitted by nonlinear regression to the candidate isotherms. The model parameters were obtained by minimizing the sum of squared errors between predicted (*q_cal_*) and experimental data (*q_exp_*). The sum of squared errors is given by:

(6) $$SE=\overset{N}{\underset{i=1}{\sum }}{\left(q_{cal}-q_{exp}\right)}_{i}^{2}$$

In either case, a plot of *c_eq_* versus *q_eq_* generated the experimental isotherm curves. For a detailed description of the adsorption isotherm models, see Supplementary Materials.

### 3.7. Isotherm Model Selection for Adsorption Equilibrium Description

The Akaike information criterion evaluates the relative adequacy of models to predict the experimental data, and thus provides a measure for the selection of the best-fit model [28]. The statistical parameters of the AIC necessary for model selection are briefly presented hereafter. A more detailed description and their mathematical derivation is given elsewhere [28]. The AIC value can be generally defined as:

(7) AIC=−2ln(L)+2K

where *L* is the maximized likelihood function for the respective model and *K* is the number of parameters in this model.

Assuming that the model errors are normally and independently distributed, and incorporating the least squares estimation, the AIC transforms to:

(8) $$\mathrm{AIC}=N\;ln\left(\frac{SSE}{N}\right)+2K$$

where *N* is the number of observations and *SSE* is the sum of squared errors.

The *SSE* is calculated analogous to Equation (6) for the difference between predicted (*q_eq_,_cal_*) and experimental adsorbent loading (*q_eq,exp_)* at equilibrium. For the case that *K* is large relative to *N* (*N/K* < 40), Sugiura [46] conceived the so-called corrected Akaike information criterion (AIC_c_), which is expressed as:

(9) $${\mathrm{AIC}}_{c}=N\;ln\left(\frac{SSE}{N}\right)+2K+\frac{2K\left(K+1\right)}{N-K-1}$$

The individual AIC and AIC_c_ scores lack universal comparability, as they are influenced by sample size, for instance, and must be converted for a meaningful interpretation of model plausibility as follows:

(10) $$\Delta_{i}={\mathrm{AIC}}_{c_{i}}-{\mathrm{AIC}}_{c_{min}}$$

where Δ*_i_* is the information loss occurring if model *i* is selected rather than the best-fit model, *i_min_*, for conclusions. AIC_c*i*_ and AIC_c*min*_ present the AIC_c_ value for model *i* and the minimum AIC_c_ value of all models studied, respectively. Hence, the best approximating model yields Δ*_i_* = 0 and with increasing Δ*_i_*_,_ the strength of evidence for the respective model decreases. The transformation $L\;\left(model/data\right)\;\propto \;\exp\;\left(-\frac{1}{2}\Delta_{i}\right)$ facilitates an estimation of the likelihood of model *i*, where $\exp\;\left(-\frac{1}{2}\Delta_{i}\right)$ is the relative likelihood of model *i*, and therefore permits an evaluation of the probability of a model being the most approximating among all candidates. The normalization of the relative likelihoods yields the Akaike weight, reflecting the “weight of evidence” in support of a model to be the best fitting, and is defined as:

(11) $$w_{i}=\frac{e^{\left(-\frac{1}{2}\Delta_{i}\right)}}{\sum_{i=1}^{R}e^{\left(-\frac{1}{2}\Delta_{i}\right)}}\;$$

where *w_i_* is the Akaike weight of the *i*th model [28].

Additionally, the evidence ratio (*ER*) quantifies how much more likely the best approximating model is than model *i*, and is given by:

(12) $$ER_{i}=\frac{w_{best}}{w_{i}}$$

where *w_best_* is the Akaike weight of the best model [28].

### 3.8. External Mass Transfer Model (EMTM)

In-depth investigations of the adsorption kinetics were conducted to elucidate the governing mechanism and characteristics of the adsorption processes studied. We applied the external mass transfer model (EMTM) to describe the decay curves of TMP and ATN adsorption on PPH [21]. The EMTM supposes that the rate of adsorption is limited solely by the external mass transport through the boundary layer. The model assumes the absence of a concentration gradient inside the particle, and therefore that intraparticle diffusion occurs instantaneous. Hence, the model excludes the presence of an internal mass transfer resistance. The EMTM and the initial and boundary conditions [29] are given by:

(13) $$\frac{dc}{dt}=-A_{S}k_{F}\left(c-c_{r}\right)$$

(14) $$t=0,\;c=c_{0}$$

(15) $$\frac{\varepsilon_{p}}{\rho_{p}}\frac{\partial c_{r}}{\partial t}+\frac{\partial q}{\partial t}=\frac{A_{S}k_{F}}{m}\left(c-c_{r}\right)$$

(16) $$t=0,\;c_{r}=0,\;q=0.$$

Here, *c* and *q* are the adsorbate concentration in aqueous solution (μg/L) and on the adsorbent (μg/g), respectively; *c_r_* is the adsorbate concentration inside the particle (μg/L); *m* is the mass of the adsorbent (g); *A_S_* is the total external surface for mass transfer (1/cm); *ε_p_* is the adsorbent particle porosity; *ρ_p_* is the apparent density of the adsorbent (g/cm^3^); *k_F_* is the external mass transfer coefficient through the boundary layer (cm/s).

The total external surface of the adsorbent particle available for mass transfer resulted from:

(17) $$A_{S}=\frac{6M}{d_{p}\rho_{p}\left(1-\varepsilon_{p}\right)},$$

where *M* is the adsorbent mass concentration in solution (g/mL) and *d_p_* is the adsorbent particle diameter (cm) [47].

From Equation (3), the derivative in time of q becomes:

(18) $$\frac{\partial q}{\partial t}=\frac{\partial q}{\partial c_{r}}\frac{\partial c_{r}}{\partial t}=K_{H}\frac{\partial c_{r}}{\partial t},$$

therefore, Equation (15) above can be rewritten as:

(19) $$\left(\frac{\varepsilon_{p}}{\rho_{p}}+K_{H}\right)\frac{\partial c_{r}}{\partial t}=A_{S}k_{F}\left(c-c_{r}\right).$$

In this case, the ordinary differential equations of the EMTM can be exactly solved with the given initial conditions. The concentration in aqueous solution changes with time as:

(20) $$c\left(t\right)=c_{0}\frac{\left(1+e^{-A_{s}{\;k}_{F}\;t\left(\frac{M\lambda +1}{M\lambda }\right)}M\lambda \right)}{1+M\lambda },$$

where

(21) $$\lambda =\left(\frac{\varepsilon_{p}}{\rho_{p}}+K_{H}\right).$$

The final *k_F_* values were determined by minimizing the nonlinear least squares objective function between predicted data (*c_t_,_cal_*) from Equation (20) and experimental data (*c_t_,_exp_*) of TMP or ATN concentrations and were calculated analogous to Equation (6).

### 3.9. Adsorption Thermodynamics

Detailed investigations of the adsorption thermodynamics are crucial to derive information on the nature and mechanism of the adsorption process. The relevant thermodynamic parameters considered for this evaluation, i.e., the standard free Gibbs energy (Δ*G*°), standard enthalpy (Δ*H*°), and standard entropy (Δ*S*°), were computed according to following equations [18,19]:

(22) $$\Delta G^{{}^{\circ}}=-RTln\left(K_{eq}\right),$$

where *R* is the universal gas constant (8.3145 J/mol K), *T* is the temperature (K), and *K_eq_* represents the thermodynamic equilibrium constant (-). Δ*G*° was derived directly from Equation (22). The relationship of Δ*G*° with Δ*H*° and Δ*S*° is given by:

(23) $$\Delta G^{{}^{\circ}}=\Delta H^{{}^{\circ}}-T\Delta S^{{}^{\circ}}.$$

Substitution of Equation (22) into Equation (23) results in the Van’t Hoff equation, which is expressed as:

(24) $$ln\left(K_{eq}\right)=\frac{\Delta S^{{}^{\circ}}}{R}-\frac{\Delta H^{{}^{\circ}}}{RT}.$$

The plot of *lnK_eq_* versus *10^3^/T*, known as the Van’t Hoff plot, facilitates the determination of Δ*H*° and Δ*S*° from the slope and intercept, respectively [29]. A correct calculation of *K_eq_* is imperative for a meaningful estimation of the thermodynamic parameters. The dimensionality and logarithmic computation in Equations (22) and (24) require *K_eq_* to be dimensionless [18,19]. In case the equilibrium data is described by the Henry isotherm, the thermodynamic equilibrium *K_eq_* constant may be obtained from the isotherm constant *K_H_* after appropriate conversion. The conversion of *K_H_* allows omitting the dimensions and is given by:

(25) $$K_{eq}\approx K_{H}\rho_{W}\left(T\right),$$

where *ρ_W_* is the density of water (g/L) at temperature *T* [19].

### 3.10. Statistical Analysis

All experiments were conducted in triplicate and the data were expressed as mean values ± standard deviation unless otherwise stated. One-way analysis of variance (ANOVA) with Tukey’s post-hoc test was carried out to compare mean group values. An alpha value of 0.05 was considered to be significant. The data was analyzed using Origin, Version 2021 (OriginLab Corporation, Northampton, MA, USA).

## 4. Conclusions

Pigeon pea husk (PPH) demonstrated encouraging properties as a low-cost adsorbent for the removal of two abundantly occurring pharmaceuticals in Indian water bodies, namely trimethoprim (TMP) and atenolol (ATN). PPH exhibited a linear increase in adsorption capacity upon increasing concentrations of TMP and ATN considering the order of magnitude of actual concentrations of these pharmaceuticals found in Indian waters. Additionally, the description of the saturation limit with an adequate isotherm model, selected based on the AIC criterion, validated the technological suitability of PPH for the studied adsorbates likewise at a higher concentration. The investigations of the adsorption kinetics using the EMTM revealed coherently that film diffusion posed the exclusive mass transfer resistance of TMP or ATN adsorption onto PPH. Furthermore, thermodynamic elucidations unveiled weak molecular interactions as driving mechanisms for both adsorption systems studied, and hold promise for future work on the regeneration of PPH and the desorption of the adsorbates. The highest adsorption loadings achieved for TMP and ATN concur with the pH range reported for Indian water bodies, substantiating PPH as a viable asset for low-cost adsorption of the considered pharmaceuticals. This study provides proof of concept of the merits of PPH as a low-cost adsorbent for TMP and ATN and depicts the promising prospect of extending its application to contaminants with similar characteristics. Ultimately, our findings depict the scientific foundation for future work on multicomponent systems, the investigation of a column adsorber design for practical applications, and the reusability of PPH aiming at the development of a viable adsorber system for low-cost water treatment.

## Figures and Tables

**Table 1:** Physical properties of PPH adsorbent material.

Physical Properties	PPH
Specific surface area (m^2^/g)	0.21
Particle size (μm)	74
Apparent density (g/cm^3^)	0.72
Solid density (g/cm^3^)	1.5
Particle porosity (-)	0.52

**Table 2:** Results of model selection among five isotherm models based on AIC for adsorption equilibrium modeling and Langmuir parameters derived for TMP or ATN adsorption onto PPH.

	Freundlich	Redlich-Peterson	Sips	Tóth	Langmuir		
					*q_mL_* (mg/g)	*K_L_* (L/mg)	*R_L_*
TMP					22.7	0.04	0.06–0.5
*K*	2	3	3	3	2		
AIC	33	9	9	9	7		
AIC_c_	34	10	10	10	8		
Δ*_i_*	26	2.5	1.8	1.7	0.0		
*w_i_*	1.02 × 10^−6^	0.14	0.19	0.20	0.47		
*ER*	461643	3	2	2	1		
					*q_mL_* (mg/g)	*K_L_* (L/mg)	*R_L_*
ATN					29.8	0.054	0.06–0.48
*K*	2	3	3	3	2		
AIC	41	−12	−12	−12	−14		
AIC_c_	42	−11	−11	−11	−14		
Δ*_i_*	56	2.5	2.4	2.4	0.0		
*w_i_*	4.4 × 10^−13^	0.15	0.16	0.16	0.53		
*ER*	1.2 × 10^12^	3	3	3	1		

**Table 3:** Comparison of maximum adsorption capacities for TMP or ATN with various adsorbates at neutral pH and RT.

Adsorbent	Adsorbate	Isotherm Model	*q_mL_* (mg/g)	Reference
Wood chippings	TMP	Langmuir	8.3	[31]
Carbonized sewage sludge/fish waste	TMP	Langmuir	90	[32]
Montmorillonite KSF	TMP	Langmuir	130	[33]
PPH	TMP	Langmuir	22.7	This study
Kaolinite	ATN	Langmuir	10.7	[34]
Granular activated carbon	ATN	Langmuir	18.8	[35]
Activated palm kernel shell	ATN	Langmuir	192	[36]
PPH	ATN	Langmuir	29.8	This study

**Table 4:** Characteristic thermodynamic parameters of the adsorption process of TMP and ATN on PPH.

	Δ*H*° (kJ/mol)	Δ*S*° (J/(mol × K))	Δ*G*° (kJ/mol)				
			277 K	298 K	313 K	323 K	333 K
TMP	−17.3	7.1	−19.1	−19.4	−19.8	−19.6	−19.4
ATN	−16.7	3.7	−17.4	−18	−18.1	−18	−17.5

## Data Availability

The data presented in this study are available on request from the corresponding author.

## Supplementary Materials

The following are available online, Figure S1: Particle size distribution of PPH, Table S1: Effect of solution temperature on adsorption capacity of PPH for TMP or ATN. Different letters denote significant differences between isotherm points (*p* < 0.05, *n* = 3), Figure S2: Determination of point of zero charge of PPH. Description of adsorption isotherm models.

## Sample Availability

Samples of the compounds are available from the authors.
