# Supplementary material for: Pigeon Pea Husk for Removal of Emerging Contaminants Trimethoprim and Atenolol from Water
Source: Molecules. 2021 May 25;26(11):3158. doi: 10.3390/molecules26113158 (PMC8197987; doi:10.3390/molecules26113158)
Supplement: Supplementary file 1 [file molecules-26-03158-s001.zip › molecules-1223081-supplementary.pdf]

# Supplementary Materials: Pigeon pea husk for Removal of Emerging Contaminants Trimethoprim and Atenolol from Water

**Severin Eder [1], Manuel Torko [1], Alessia Montalbetti [1], Paride Azzari [2] and Laura Nyström [1,*]**

[1] Laboratory of Food Biochemistry, Institute of Food, Nutrition and Health, Department of Health Science and Technology, ETH Zurich, Schmelzbergstrasse 9, 8092 Zurich, Switzerland; severin.eder@hest.ethz.ch (S.E.); mtorko@student.ethz.ch (M.T.); malessia@student.ethz.ch (A.M.)
[2] Laboratory of Food and Soft Materials, Institute of Food, Nutrition and Health, Department of Health Science and Technology, ETH Zurich, Schmelzbergstrasse 9, 8092 Zurich, Switzerland; paride.azzari@hest.ethz.ch
* Correspondence: laura.nystroem@hest.ethz.ch; Tel.: +41-44-632-91-65

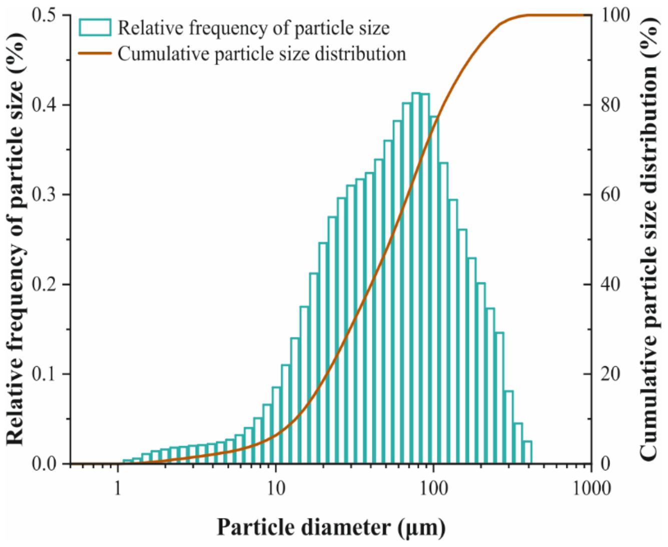


**Figure S 1.** Particle size distribution of PPH.

**Table S 1.** Effect of solution temperature on adsorption capacity of PPH for TMP or ATN. Different letters denote significant differences between isotherm points ($p < 0.05$, $n = 3$).

| TMP | | | | | |
|---|---|---|---|---|---|
| $C_0$ [ppb] | Temperature [K] | | | | |
| | 277 | 298 | 313 | 323 | 333 |
| 10 | 53 ± 7[a] | 20 ± 1[b] | 26 ± 6[b] | 20 ± 10[b] | 16 ± 6[b] |
| 20 | 90 ± 10[a] | 41 ± 6[b] | 37 ± 6[b,c] | 26 ± 6[b,c] | 22 ± 6[c] |
| 35 | 120 ± 7[a] | 72 ± 7[b] | 62 ± 6[b] | 42 ± 6[c] | 30 ± 10[c] |
| 50 | 145 ± 12[a] | 99 ± 11[b] | 89 ± 11[b,c] | 67 ± 6[c,d] | 53 ± 6[d] |
| 75 | 205 ± 15[a] | 141 ± 15[b] | 126 ± 16[b,c] | 90 ± 10[c,d] | 72 ± 6[d] |
| 100 | 278 ± 13[a] | 210 ± 21[b] | 174 ± 16[b] | 119 ± 17[c] | 97 ± 12[c] |
| 125 | 345 ± 19[a] | 244 ± 15[b] | 202 ± 17[b,c] | 166 ± 17[c] | 116 ± 17[d] |
| 175 | 487 ± 16[a] | 340 ± 40[b] | 278 ± 25[c] | 227 ± 21[c,d] | 177 ± 22[d] |
| 200 | 560 ± 40[a] | 392 ± 16[b] | 327 ± 21[b,c] | 267 ± 29[c,d] | 211 ± 29[d] |
| ATN | | | | | |
| $C_0$ [ppb] | Temperature [K] | | | | |
| | 277 | 298 | 313 | 323 | 333 |
| 50 | 103 ± 7[a] | 76 ± 5[b] | 55 ± 8[c] | 43 ± 6[c] | 23 ± 6[d] |
| 75 | 129 ± 12[a] | 113 ± 12[a] | 78 ± 4[b] | 63 ± 7[b] | 36 ± 6[c] |
| 100 | 190 ± 18[a] | 136 ± 8[b] | 99 ± 8[c] | 80 ± 11[c,d] | 60 ± 6[d] |

| | | | | | |
|---|---|---|---|---|---|
| 150 | 240 ± 21[a] | 172 ± 16[b] | 139 ± 11[b,c] | 113 ± 12[c] | 72 ± 7[d] |
| 200 | 354 ± 23[a] | 235 ± 18[b] | 190 ± 18[c] | 134 ± 16[d] | 113 ± 6[d] |
| 250 | 384 ± 17[a] | 303 ± 26[b] | 230 ± 18[c] | 182 ± 16[d] | 128 ± 10[e] |
| 300 | 467 ± 16[a] | 360 ± 20[b] | 282 ± 6[c] | 201 ± 20[d] | 155 ± 11[e] |
| 350 | 563 ± 27[a] | 443 ± 21[b] | 326 ± 21[c] | 267 ± 13[d] | 180 ± 11[e] |
| 400 | 630 ± 50[a] | 480 ± 30[b] | 377 ± 16[c] | 299 ± 19[d] | 201 ± 17[e] |

The point of zero charge (pzc) is defined as the pH value at which the net charge of the adsorbent`s surface equals zero. The pzc of Pigeon pea husk (PPH) was determined according to the pH drift method described by Khan and Sarwar [1]. In brief, a supporting electrolyte solution, consisting of 0.01 M NaCl, was prepared over a pH range from 2 to 12. The pH of the solution was adjusted with HCl or NaOH at appropriate concentration to minimalize volume distortion. Subsequently, 0.2 g of PPH were added to each 0.01 M NaCl solution adjusted to a specific pH. The mixtures were incubated in a Unimax 1010 shaker (Heidolph, Schwabach, Germany) under constant agitation at 300 rpm for 24h. Ultimately, the difference between final and initial pH (ΔpH) was plotted against the initial pH. The initial pH at which the ΔpH is zero, corresponds to the pzc of PPH.

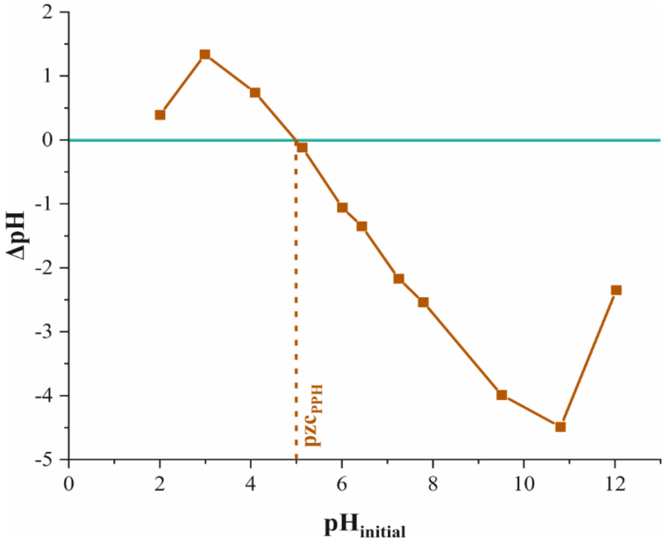


**Figure S 2.** Determination of point of zero charge of PPH.

Adsorption isotherm models

The Freundlich isotherm represents multilayer adsorption occurring on a heterogeneous surface with energetically nonuniform adsorption sites [2]. The Freundlich isotherm is presented by

$$q_{eq} = K_F c_{eq}^{1/n},$$

where $K_F$ is the Freundlich constant related to the adsorption strength ((mg/g)(L/mg)$^{-1/n}$). The Freundlich exponent $1/n$ describes the surface heterogeneity in terms of energy. The Freundlich exponent corresponds to the isotherm characteristic, being either favorable ($0 < 1/n < 1$), unfavorable ($1/n > 1$), linear ($1/n = 1$), or irreversible ($1/n = 0$).

The Redlich-Peterson isotherm incorporates elements of the Langmuir and Freundlich isotherms. Its applicability to approximate the equilibrium adsorption data on homogenous or heterogeneous surfaces

extends over a wide range of concentration. The model reduces to the Freundlich model at high adsorbate concentration ($a_R c_{eq}^g \gg 1$), hence presenting no saturation limit, and approaches the linear Henry isotherm at vanishing concentrations ($a_R c_{eq}^g \ll 1$). The Redlich-Peterson isotherm can be written as

$$q_{eq} = \frac{K_R c_{eq}}{1 + a_R c_{eq}^g},$$

where $K_R$ and $a_R$ present the Redlich-Peterson constants, (L/g) and (L/mg), respectively. The Redlich-Peterson exponent $g$ correlates with the surface heterogeneity and lies between 0 and 1. The exponent $g$ equals 1 in case of a homogenous surface, and deviates more from unity, the more heterogeneous the adsorbent surface is [3].

The Sips isotherm combines features of the Langmuir and Freundlich isotherm models to predict adsorption processes in heterogeneous systems. Hereby, it describes the complete monolayer adsorption characteristic of the Langmuir isotherm at high adsorbate concentrations ($K_S c_{eq}^{\beta S} \gg 1$) and overcomes constraints associated with the Freundlich model. Conversely, the Sips isotherm approaches the Freundlich model towards low adsorbate concentrations ($K_S c_{eq}^{\beta S} \ll 1$) [3]. The Sips isotherm is given by

$$q_{eq} = \frac{q_{mS} K_S c_{eq}^{\beta S}}{1 + K_S c_{eq}^{\beta S}},$$

where $q_{mS}$ depicts the Sips constant related to maximum monolayer adsorption capacity (mg/g). $K_S$ and $\beta_S$ represent the Sips isotherm constant (L/g) and the heterogeneity factor, respectively

The Tóth isotherm illustrates a modification of the Langmuir isotherm intended to improve the approximation of the experimental data and satisfies both boundary conditions of adsorbate concentration. The Tóth model comprises the saturation limit at high concentrations ($(K_T c_{eq})^{nT} \gg 1$), and shows a reduction to the linear form at low concentration ($(K_T c_{eq})^{nT} \ll 1$). It is commonly applied for adsorption on heterogeneous surfaces and implicates asymmetrical quasi-Gaussian energy distribution of adsorption sites, where most sites having an adsorption energy lower than the maximum. The Tóth isotherm is given by

$$q_{eq} = \frac{q_{mT} K_T c_{eq}}{\left[1 + \left(K_T c_{eq}\right)^{nT}\right]^{1/nT}},$$

where $q_{mT}$ is the Tóth constant related to maximum monolayer adsorption capacity (mg/g). $K_T$ and $n_T$ reflect the Tóth isotherm constant (L/g) and the heterogeneity factor, respectively [3, 4].


1. Khan, N. M.; Sarwar, A., Determination of Points of Zero Charge of Natural and Treated Adsorbents. *Surface Review and Letters* **2007,** 14, (03), 461-469.
2. Piccin, J. S.; Cadaval, T. R. S. A.; de Pinto, L. A. A.; Dotto, G. L., Adsorption Isotherms in Liquid Phase: Experimental, Modeling, and Interpretations. In *Adsorption Processes for Water Treatment and Purification*, Bonilla-Petriciolet, A.; Mendoza-Castillo, D. I.; Reynel-Ávila, H. E., Eds. Springer International Publishing: Cham, 2017; pp 19-51.
3. Al-Ghouti, M. A.; Da'ana, D. A., Guidelines for the use and interpretation of adsorption isotherm models: A review. *J. Hazard. Mater.* **2020**, 122383.
4. Worch, E., Adsorption equilibrium I: General aspects and single-solute adsorption. In *Adsorption Technology in Water Treatment, Fundamentals, Processes, and Modeling*, De Gruyter: Berlin, Boston, 2012; pp 41-76.
